# Editorial–A Milestone in the Technological Development of Hadrontherapy, in FranceHadrontherapy, cryogenic cyclotron

**DOI:** 10.1016/j.ijpt.2025.101288

**Published:** 2025-11-26

**Authors:** Jean-Louis Habrand, Gabriel Gaubert, Siamak Haghdoost, Juliette Thariat, Roman Rouzier

**Affiliations:** Radiation Oncology Department, François Baclesse Cancer Center, 14076, Caen, France; Directorate General, CYCLHAD Particle Therapy Center, 14200 Hérouville-Saint-Clair, Caen, France; Université Caen Normandie, Univ Rouen Normandie, Normandie Univ, ABTE UR4651, F-14000 Caen, France; Radiation Oncology Department, François Baclesse Cancer Center, 14076, Caen, France; Directorate General, François Baclesse Cancer Center, 14076, Caen, France

The symposium “Hadrontherapy for Life,” which was held in Caen, France, on March 10 and 11, 2025, was to mark the end of the assembly work of the highly innovative accelerator “C400″ for research and treatment in hadrontherapy. Co-organized by the University of Caen-Normandy, the Proton Therapy Center “CYCLHAD,” the François Baclesse Comprehensive Cancer Center and the Normandy Region, it was held at the University of Caen-Normandy, campus one, and hosted 150 or so experts from the international community.

The objective of this symposium was primarily educational in synthetizing the experience of the international community (mainly Japanese and European) in the clinical (but not exclusively) setting and further disseminate data through published reports. You might enjoy some of them in this issue. It was also a unique opportunity to unveil the prototype of a new concept of accelerator for particle radiotherapy, a rare event in a scientist or physician career!.

This equipment, the “C400″ superconducting cyclotron for heavy ions is not exactly a newcomer. Making a long story short, the concept was in the air since 1987 when the EULIMA European initiative chaired by its French designer Pierre MANDRILLON, a physicist and engineer trained at CERN, Head of the Cyclotron Laboratory in Nice, conducted a comparative investigation between this elegant approach and the more conventional synchrotron.^1^ Despite its attractiveness (esp. in terms of compacity) and due to the formidable technological challenge it represented at that time, the latter was favored although no concrete project came true.^2^ The misfortune of EULIMA benefited national programs, all based on synchrotrons in Germany (HIT, Heidelberg and MIT, Marburg), Austria (MedAustron, Wiener Neustadt), Italy (CNAO, Pavia), and France (ETOILE, Lyon). Meanwhile, Yves JONGEN, Pierre MANDRILLON’s former colleague, physicist, engineer, founder and CEO of the Belgian IBA proton therapy company had revisited the “C400″ design and its feasibility, a collaborative effort with the physics group at JINR-Dubna.^3^ But the mountain was still too high for a single mountaineer to climb, and the wind turned again: the ETOILE project, short of money, was shut down in the mid-2010s. A competitor was born at the GANIL nuclear research center in Caen in 2003 with an attractive project mixing physicists, engineers, biologists, and eventually clinicians under the acronym of ASCLEPIOS, and later ARCHADE. Importantly, this project was strongly supported by the University and financed by the governance of the region of Normandy, which is sort of Mecca for the French civil and military nuclear industry since the early 70 seconds. The saga approaches the end with the creation by the Normandy-Region of a multinational consortium of French, Belgian (of course!) and other European industrials and stakeholders supported by European banks: The *Normandy-Hadrontherapy* group.

Let’s introduce you the final design of “C400″: a 400 MeV/u cryogenic isochronous cyclotron, compact and implantable in a confined environment (ie, hospital-based) with its 3.5 m radius (less than a third of a conventional synchrotron), able to produce a wide range of heavy particles (at least from protons to carbon ions) at clinically relevant beams energy, intensity, and size, and supposedly available on the market at reasonable price and running cost. It will join at the CYCLHAD Proton Therapy Center, an existing “ProteusONE” single-room solution for proton-therapy (PT). As far as we know, no alternative, technological or commercial, is available worldwide.

The original program at the CYCLHAD Center itself dates back 2011. The idea behind was to initiate on a piece of land released by GANIL a limited PT single-room solution program for a maximum capacity of 345 patients (10 500 fractions or so) equipped with spot scanning technology and gantry, as a sort of “dummy run” for further investigations dealing with heavier ions. A positive impact on the overall budget balance was also expected, although it turned out to be below expectations in the long run. All patients could be worked up and simulated at the François Baclesse Comprehensive Cancer Center, located nearby. A pediatric program was also implemented in collaboration with the Caen University Hospital. Importantly, this approach managed time for the “C400″ technical constraints to mature. Ramp-up of the clinical program may start in 2028 to a maximum capacity of 7500 or so additional sessions, eventually connected to 3 rooms for physical, biological, and clinical investigations. It should be open to national and international patients, researchers, and industrials. The former PT program will go on in parallel, and a radiobiology platform with in-transit animal housing will complete the offer at an upper level of the CYCLHAD building. This design should be attractive for low and high LET particles pre-clinical and clinical inter-comparisons and end up with financial balance hopefully!.

First “C400″ beams are expected mid-2026, by the time of the next annual “PTCOG 64″ congress in Caen/Deauville. We bet that the first “baby’s cry” will attract much attention from our community, including students and young staff, and represent a milestone in the development and education of hadrontherapy.

## CRediT authorship contribution statement

**J.L. Habrand:** Conceptualization, Writing – original draft preparation, Funding acquisition. **G. Gaubert:** Conceptualization, Writing – reviewing and editing, Funding acquisition. **S. Haghdoost:** Conceptualization, Writing – original draft preparation. **J. Thariat:** Conceptualization, Writing – original draft preparation. **R. Rouzier:** Conceptualization, Writing – reviewing and editing, Funding acquisition.

## Declaration of Competing Interest

The authors declare that they have no known competing financial interests or personal relationships that could have appeared to influence the work reported in this paper.
